# Use of the Mental Health Gap Action Programme (mhGAP) by mental health volunteers in the UK: examples from South Asian diaspora charities

**DOI:** 10.1192/bjb.2024.116

**Published:** 2026-02

**Authors:** Leya Luhar, Aditi Arya, Raeesah Rafiq, Nandini Chakraborty

**Affiliations:** 1 Leicester Medical School, University of Leicester, Leicester, UK; 2 University Hospitals of Leicester NHS Trust, Leicester, UK

**Keywords:** Community mental health teams, education and training, mental health services, stigma and discrimination, transcultural psychiatry

## Abstract

The Mental Health Gap Action Programme (mhGAP) was launched by the World Health Organization (WHO) in 2008 to scale up services for mental, neurological and substance use disorders for low- and lower-middle-income countries. Subsequently, an updated mhGAP intervention Guide (mhGAP-IG 2.0) was released in 2016. This study explores the use and effectiveness of mhGAP-IG 2.0 by mental health volunteers of two South Asian charities in the UK. Semi-structured interviews were carried out with eight volunteers. The core themes identified were mental health awareness, mental health education, empathy and care, social perception and bias within the South Asian community, and personal development. The study identified mhGAP as a tool with transformative potential. Although the WHO originally planned the mgGAP-IG as a tool for low- and middle-income countries with limited mental health resources, this study demonstrates its usefulness even in high-income countries, as a foundation to educate volunteers working in mental health.

In 2008, the World Health Organization (WHO) launched the Mental Health Gap Action Programme (mhGAP), with the aim of scaling up services for mental, neurological and substance use disorders for countries, particularly with low- and lower-middle incomes.^1^ The programme asserts that with appropriate care, psychosocial assistance and medication, tens of millions of people with high-burden diseases, such as depression, schizophrenia and epilepsy, could be effectively treated, protected from suicide and empowered to achieve a high quality of life – even in settings where resources are scarce. Fifteen years and two subsequent iterations later,^2^ the mhGAP has been extensively applied and studied in several global settings, demonstrating a considerable impact on healthcare services worldwide, with use in over 100 countries.^3^ However, its application and role in higher-income countries, such as the UK, particularly within minority communities, has remained largely unexplored, despite the advocation for its use.^4^

In 2016, the WHO released an updated mhGAP Intervention Guide (mhGAP-IG 2.0), which provides evidence-based guidance and tools for the assessment and integrated management of priority mental, neurological and substance use disorders,^5^ using clinical decision-making protocols.^5^ This article explores an innovative utilisation of the mhGAP-IG 2.0 by two South Asian charities in the UK, both dedicated to raising mental health awareness. Their use of the mhGAP-IG 2.0 is designed to equip community volunteers with training to provide appropriate psychosocial assistance to patients in contextual cultural settings. Although it may seem to diverge from its original purpose, the need to scale up services and resources during a time of significant strain on the National Health Service (NHS) aligns with the core mission of the mhGAP. As a well-validated, field-tested tool of international repute, the mhGAP is designed to accommodate cultural differences, making it suitable for training volunteers in diverse settings. Relevant adaptations were made when training volunteers on the use of the mhGAP, by emphasising the assessment algorithms and guiding volunteers to identify when signposting was appropriate, and to which organisations.

Through a qualitative approach, conducting semi-structured interviews with charity founders and volunteers, we analysed how the mhGAP's guidelines and training resources can be adapted and applied in an unconventional setting. This adaptation has the potential to significantly extend mental health support reach, particularly by leveraging an invaluable resource: charity volunteers. This strategy not only addresses the unique needs of minority communities, but also presents as a supplementary solution to alleviate the pressures on the UK's overstretched healthcare system. As of October 2023, the UK had 4485 full-time equivalent consultant psychiatrists^6^ for a population of approximately 67.7 million people, which translates to one consultant psychiatrist per 15 000 people.^6^ In addition to these statistics, studies have evidenced the presence and persistence of ethnic inequalities in accessing mental healthcare in the UK, exacerbating the challenge of providing adequate support.^7^

## South Asian Health Action

South Asian Health Action (SAHA), established in 2017 as a national charitable organisation, aspires to be the UK's leading patient and community-led health and social care charity. SAHA is committed to engage with, educate, explore and empower South Asian communities^8^ on the specific health and social care challenges that they face disproportionately compared with the general population. This understanding is enriched because of the anecdotal experiences of its volunteers and the founder, Kirit Mistry, who often share personal encounters with the very issues SAHA aims to address, adding a personal and insightful dimension to SAHA's approach. SAHA adopts a focused strategy to tackle these issues in a more individual manner, critically addressing the limitations of projects broadly labelled as ‘BAME-targeted’ that often fail to recognise the nuances between different communities. SAHA's focus on South Asian communities emerged because of Mistry's recognition of the need for culturally adapted services, which considered dietary preferences, deep-rooted traditional practices and linguistic diversity within these communities.^9^

SAHA has adapted existing resources to develop checklists to evaluate the severity of an individual's depression or anxiety, coupled with toolkits for referring them to the necessary services. Another unique strength of SAHA's lies in its network of medical advisors from various specialties within the target communities, enabling the provision of customised guidance and support. This expertise is showcased in a variety of events tailored to the community's needs, such as lifestyle and management sessions with healthcare professionals from India, yoga sessions for Diabetes Awareness Week led by specialist instructors, and foot and diabetes care sessions conducted by specialist diabetes podiatrists. These initiatives reflect SAHA's comprehensive approach to health and wellness, emphasising the importance of culturally sensitive and accessible healthcare solutions.^9^

## The Sangeet Foundation

The Sangeet Foundation (registered as Sangeet Global Limited) is another registered charity in the UK that applies a unique approach within the South Asian community, championed by its ethos of ‘happiness through music and the arts’, as their objective is to showcase the power of music and the arts as a complementary mental health therapy. Since its formation in 2015, the Sangeet Foundation has been a beacon of cultural celebration, primarily focusing on live musical concerts. To date, they have successfully organised and hosted more than 100 events across online platforms and in-person workshops.^10^ These events feature renowned performing artists alongside mental health professionals such as psychiatrists, psychologists and counsellors, encouraging an open dialogue on mental health.

Founded by Jayanta and Nupur Ray, this initiative was inspired by their own experience with their daughter, a student who was diagnosed with adult attention-deficit hyperactivity disorder, anxiety and dyspraxia. Initially struggling with acceptance and attributing her challenges to what they perceived as Western misconceptions – arising from the stigma surrounding mental health in South Asian cultures – they were reluctant to embrace the diagnosis and concerned about societal judgement. However, witnessing the profound impact of music and meditation on their daughter's mental well-being led them to a pivotal realisation about the healing power of the arts and the importance of abolishing the longstanding taboo of mental health issues in traditional households.^10^

## Aims

The aim of our research was to examine the deployment of the mhGAP-IG 2.0 by non-medical professionals, to support members of the South Asian diaspora in the UK who require mental health assistance. By doing so, we aspire to contribute to the long-term ideal of reducing cultural health disparities and enhancing the provision of mental health support in the UK.

Volunteers from both charities had received training in mhGAP IG 2.0 from Professor Nandini Chakraborty over two sessions. through a combination of face-to-face and online interactions. and had been using their skills to help members of their communities understand mental health conditions and seek help at the right time and place.

## Method

Semi-structured interviews were carried out with eight volunteers from both SAHA and the Sangeet Foundation, to explore the important elements in the role of a volunteer and the contribution of the mhGAP to their effectiveness. Participants gave verbal consent; the interviews were recorded, and participants were informed that they could withdraw consent at any point.

## Results

A thematic analysis summarised in Table 1 demonstrates the results of the study, which were categorised under five themes*:* mental health awareness, mental health education, empathy and care, social perception and bias within South Asian communities, and personal development.

**Table 1:** Thematic analysis of volunteer interviews

Themes	Codes	Example quotations	*n* (participants)
Empathy and care	Support for individuals and families Personal experience Emotional impact	‘ … listening empathetically, unbiased, and being non-judgemental … ’ ‘ … providing emotional support … ’ ‘ … it is imperative that the family members of those suffering from mental health issues are allowed to share their experiences too without being prejudiced or judgmental’ ‘The power of storytelling and using your own lived or living experience to help others … ’	8
Mental health awareness	Spreading awareness Publicity	‘ … work towards spreading the awareness of mental health in the community’ ‘Volunteers may engage in activities such as raising awareness … ’ ‘ … create targeted campaigns and digital channels … ’ ‘promote more widely on various social media platforms … ’	7
Mental health education	Signposting Giving advice	‘Knowing what sources of support (are) available and signposting people to them’ ‘ … be able to offer practical tips and advice … ’ ‘ … guide them to seek help from authorised practitioners from various disciplines of mental health’	7
Social perception and bias within South Asian communities	Stigma Denial	‘ … deep seated feeling of low self-esteem within the family’ ‘There are others who refuse to accept or talk about it’ ‘ … fearful and anxious about having conversations … ’	4
Personal development	Training Competence Self care	‘ … thinking what I can be doing more of to improve but also help myself also in such situations’ ‘ … helped volunteers in understanding different mental health disorders, identifying symptoms, and providing appropriate support and referrals’ ‘A reminder to look after yourself’	3

Seven out of eight participants stated that mhGAP helped spread mental health awareness (by fostering recognition of this topic) and identified this as central to their role. One volunteer commented that the main difficulty they had faced as a volunteer was ‘no awareness of volunteer mental health services’, indicating the need to increase visibility of these services.

Another volunteer explained how the mhGAP serves also as a source of motivation, noting it apprised them with an understanding on the challenges that the world would face in the next few decades, wherein cases of mental health issues would possibly increase to alarming levels.

The strategies to promote mental health awareness included organising community events and workshops, collaborating with other organisations and stakeholders, leveraging social media and online platforms, engaging in public speaking and advocacy, and providing educational materials and resources to the public.

Seven out of eight participants stated that the mhGAP helped achieve mental health education that aims to cultivate a deeper understanding and insight into the subject, and several volunteers emphasised this to be a core aspect of their role. The observation was that ‘understanding of mental health is much more limited than physical health’.

Volunteers provided mental health education by offering ‘practical tips and advice’ to patients, particularly while they awaited specialist care. Additionally, both charities hosted forums where experts, academicians and luminaries from the field of mental health studies could discuss and share insightful information about the commendable resources available to help an individual.

All eight participants unanimously agreed that mhGAP succeeded in providing empathy and care. Volunteers often encounter the challenge of establishing trust and rapport with individuals who have had unpleasant encounters with mainstream health services. To address this, volunteers prioritise listening empathetically and creating a safe and supportive environment for individuals to share their experiences.

Four of eight volunteers recognised that mhGAP helped identify social perception and bias within the South Asian community. The stigma related to mental health often leads to ‘families suffering in silence’. Reputation in South Asian culture can present a major barrier to seeking professional help for mental health concerns,^11^ with families worrying about ‘social isolation within the community’. Volunteers expressed concerns that individuals could refrain from seeking help because of ‘fear of being judged by friends and family’.

To overcome these barriers, volunteers actively promoted open discussions, educated the community and provided safe spaces for individual conversations. The many misconceptions about mental health, including viewing conditions as ‘excuses’ or ‘attention-seeking behaviours’, were addressed. Volunteers emphasised that mhGAP, as an evidence-based resource, did lend credibility to their efforts, increasing the likelihood that people would listen and reconsider their views.

Three out of eight volunteers acknowledged that the mhGAP training significantly enhanced their competence and proficiency in mental health services, which led to their personal development. This was significant given that volunteers do not receive monetary compensation. One volunteer noted that they implement their learnings from the mhGAP for their personal use. Moreover, volunteers emphasised the role of mhGAP training in strengthening their emotional resilience. Additionally, several volunteers mentioned that the programme taught them the importance of self-care strategies in preventing burnout and maintaining overall well-being.

## Discussion

The purpose of this study was to examine the deployment of the mhGAP-IG 2.0 by non-medical professionals, to support members of the South Asian diaspora in the UK requiring mental health assistance. By conducting interviews with the volunteers of SAHA and the Sangeet Foundation, we were able to elaborate on the role of mhGAP training in providing timely care. We were also able to identify what the volunteers considered their role in using the mhGAP tool.

### SAHA and mhGAP

SAHA's primary role within the patient pathway is to mitigate the interim between a patient's referral and their consultation with a specialist. This intervention addresses the widely recognised challenges posed by the NHS's long waiting lists^12^ and constrained capacity, which often delay timely access to necessary care. Skilled volunteers within SAHA step in to provide essential emotional support to patients, serving as a source of encouragement and motivating individuals to pursue the services they require. They are also able to recommend effective coping strategies to manage their conditions in the meantime. These dedicated volunteers maintain consistent contact with patients and, when appropriate, offer signposting to additional support services.^9^

Mistry refers to SAHA as an ‘advocacy organisation’, highlighting its innovative approach to patient support, given its non-commissioned status and the inherent limitations thereof. An example of this advocacy is patients who may be reluctant to attend appointments because they feel that they are not being listened to, or that their doctor is not culturally competent, may be accompanied in their appointments by SAHA volunteers. The volunteers advocate on their behalf to ensure their concerns are addressed, thereby observing and evaluating the healthcare provider's cultural competency and patient engagement practices.^8,9^

The explicit intention behind SAHA's adoption of the mhGAP is to provide volunteers with a framework of current local healthcare pathways. This approach is designed to enable volunteers to effectively signpost patients to the most appropriate organisations for support. A notable example of this implementation was the recommendation of a 24/7 mental health services support centre in Leicester, which proved invaluable during the pandemic. The centre was previously unknown to many, and its use significantly alleviated pressure on local general practices.

SAHA places a high value on the well-being and support of its volunteers, who are integral to the organisation's mission. The organisation implements a peer pairing system among volunteers, ensuring they receive the necessary peer support to fulfil their roles effectively. Following any initial training on the mhGAP I-G 2.0, the organisation conducts bi-monthly check-ins to assess the volunteers’ application of the mhGAP I-G 2.0 in their interactions and to identify any challenges they encounter. This proactive approach enables SAHA to continuously refine and enhance its support mechanisms, ensuring that volunteers are well-equipped to navigate their responsibilities.^9^

### The Sangeet Foundation and mhGAP

The Sangeet Foundation's approach to the mhGAP differs in their intention to raise awareness and enhance the understanding of mental health conditions among the general public. Through their events and accumulated knowledge, they create an environment conducive to the open discussion of often stigmatised topics, guiding families to specialists in the community who can offer general advice, answer queries and refer them onward. An issue that the Sangeet Foundation hopes to address with its employment of the mhGAP is the incorrect or incomplete information that is propagated by unverified sources such as content creators on social media platforms. Ray clarifies that he does not intend for the volunteers of the organisation to become counsellors, but rather to further understand the intricacies of mental health and wellness, and to be the ‘first port of call’ for the people around them in need of support.^10^

Ray comments that the charity often receives enquiries from parents worried about their children, facing a similar situation to his own family. This indicates trust in the charity as a source of guidance and highlights the importance of understanding the potential issues community members may encounter in this realm. Overall, within the Sangeet Foundation, the mhGAP serves as a resource to raise mental health awareness and encourage the recognition of mental health disorders as legitimate conditions.^10^

### Volunteer perspectives

In our study, we found that the volunteers identified that the mhGAP training helped them provide mental health awareness and education, as well as benefitting them with personal development and growth. The volunteers felt compelled to spread the awareness gained from the mhGAP initiative as much as possible to their peers and other volunteers, who could then be prepared to address the more demanding needs of society at large. This perspective demonstrates the volunteers’ recognition of the critical role that informed advocacy plays in pre-empting future challenges in mental health, fuelled by insights gained from the mhGAP. The volunteers also suggested additional avenues for outreach programmes.

Volunteers identified the mhGAP as a source of mental health education, stating that it ‘provides evidence-based guidelines for the assessment and management of mental health conditions’ It is commonly regarded as a ‘reference point’, aiding volunteers in identifying appropriate courses of action to recommend and undertake themselves. The mhGAP was described as ‘easy to follow and segmented into relevant areas really well’, making it an accessible source of education. Volunteers signposted the sources of support to those needing them.

An illustrative example of utilising the mhGAP for educational purposes comes from one volunteer's experience as a secondary school teacher. Faced with students grappling with anxiety, they disseminated the mhGAP-IG 2.0 to their school faculty to enhance their understanding of addressing such cases. This exemplifies how the mhGAP serves as a catalyst for nurturing greater awareness and expertise in mental health education, both within and beyond the volunteer community.

Volunteers in SAHA and the Sangeet Foundation are deeply dedicated to providing empathy and care to individuals in the community who are affected by mental health disorders or have family members with such issues. Recognising the significance of familial involvement in patient care, volunteers approach interactions with the patient's family members with ‘empathy, respect, and a non-judgmental attitude’. This acknowledgment of family support's pivotal role in overall well-being is exemplified by the founders of the Sangeet Foundation, who often draw from their own experiences to facilitate familial reconciliation. For instance, one founder recounted an instance where they acted as mediators between a young adult and their parents. The young adult, estranged from their parents because of a strained relationship, had left home. The Sangeet Foundation intervened to support their communication and reinstate the family unit.

For many volunteers, empathy serves as a driving force behind their decision to engage in their role. One volunteer explained that many of them ‘find fulfilment in supporting the well-being of individuals in the South Asian community using their own spoken language to assist and cross the language barriers and get safe results’. Much of this empathy stems from personal experiences with their own, or close friends’ and family members’, struggles with mental health challenges. Volunteers acknowledge that their lived experiences provide them with a unique advantage in delivering care, as it enables them to establish deeper connections with patients based on shared understanding and empathy.

When asked about the role of the mhGAP in delivering care with empathy, volunteers unanimously elaborated on how the programme equipped them with comprehensive insights into the actual prevalence and complexities of mental health conditions. Gaining an understanding of the multifaceted challenges faced by individuals navigating mental health issues served as a foundation for the development of empathy in volunteers.

Many volunteers identified resistance or stigma related to mental health within the South Asian community as a common obstacle in their efforts, posing a challenge to the missions of both Sangeet Foundation and SAHA. Recognising the role of community support in the biopsychosocial treatment model,^13^ both organisations prioritise addressing these issues head-on. In their efforts to dispel the stigma and denial of the issues, volunteers actively engage in open discussions to educate the community and use the mhGAP, as an evidence-based resource, to aid their efforts and maximise their impact.

Finally, the mhGAP training also serves as a means to enhance volunteers’ skill-set – a form of compensation in itself, which has also been emphasised by Mistry of SAHA. In our study, the chosen volunteers also acknowledged that the training significantly enhanced their competence and proficiency in mental health services, which carried personal benefits such as encouraging self-care and reminding them to look after themselves so that they could better serve others.

It was intriguing to observe the diverse approaches employed by SAHA and the Sangeet Foundation in utilising the mhGAP, each proving effective in its distinct manner, for individuals seeking mental health support.

### Strengths and limitations

The limitation of our study is the small sample size, and future larger sample studies might help corroborate the insights we developed. We determined that no new themes emerged in the later interviews, indicating that thematic saturation had been reached. As a result, only eight volunteers were included in this study. However, future research in this area should involve a larger sample size, enabling more comprehensive and generalisable conclusions to be drawn from the results.

Our study was confined to exploring the effectiveness of using mhGAP in the South Asian diaspora, but we did not include experiential effects of racism, migration, religion and complex trauma, which Bansal et al found more relevant than support based on ethnic group classification alone.^7^ However, the strength of our study is that it involves lessons from local organisations such as SAHA and the Sangeet Foundation, and this is in agreement with the conclusion of Karasz et al^11^ who state that lessons from local organisations need application at a national level, to promote cultural responsiveness in treating mental illness in the South Asian immigrant community.

In conclusion, as demonstrated by SAHA and the Sangeet Foundation in our study, the mhGAP is a tool with transformative potential. As minority ethnic communities face unique cultural, social and systemic challenges, the adaptability and efficacy of the mhGAP could support an advancement in mental health support. By offering culturally sensitive and evidence-based guidelines, the programme empowers non-specialists, including dedicated volunteers, to deliver care tailored to the specific needs of communities. The results of this study highlight the potential of the mhGAP in high-income countries with diverse communities, expanding its application beyond its original purpose.

Our research also underlines the critical need for a multifaceted approach to improve mental health support across diverse communities. Healthcare providers must be called upon to enhance the cultural competence of their services, ensuring they are inclusive and effectively meet the varied needs of all populations. Funders may also play a pivotal role, by allocating resources to community organisations that have established trust and reach within their communities. These organisations are uniquely positioned to deliver alternative, informal support and serve as essential bridges between formal mental health services and the community. Communities themselves must be empowered to engage in more open and frequent discussions about mental health. Through this approach, the mhGAP holds promise in mitigating disparities in mental healthcare access and promoting greater equity in mental health support across diverse populations.

## Data Availability

The data that support the findings of this study are available from the corresponding author, N.C., upon reasonable request.

## About the authors

**Leya Luhar** was a medical student at Leicester Medical School, University of Leicester, Leicester, UK at the onset of the study. **Aditi Arya** was a medical student at Leicester Medical School, University of Leicester, Leicester, UK at the onset of the study; she is a foundation year 1 doctor at University Hospitals of Leicester NHS Trust, Leicester, UK. **Raeesah Rafiq** was a medical student at Leicester Medical School, University of Leicester, Leicester, UK at the onset of the study. **Nandini Chakraborty** is a consultant psychiatrist in Early Intervention in Psychosis, Leicestershire Partnership NHS Trust, Leicester, UK; and Honorary Professor, Department of Population Health Sciences, University of Leicester, Leicester, UK.
